# Long term extension of a randomised controlled trial of probiotics using electronic health records

**DOI:** 10.1038/s41598-018-25954-z

**Published:** 2018-05-16

**Authors:** Gareth Davies, Sue Jordan, Caroline J. Brooks, Daniel Thayer, Melanie Storey, Gareth Morgan, Stephen Allen, Iveta Garaiova, Sue Plummer, Mike Gravenor

**Affiliations:** 1 Swansea University Medical School, Singleton Park, Swansea, UK; 2 Department of Nursing, The College of Human and Health Sciences, Swansea University, Singleton Park, Swansea, UK; 3 The Children’s Trust, Tadworth, Surrey, UK; 4 The Harley Street Clinic Children’s Hospital, London, UK; 5 Liverpool School of Tropical Medicine, Pembroke Place, Liverpool, UK; 6 Research Department, Cultech Limited, Baglan Industrial Park, Port Talbot, UK

## Abstract

Most randomised controlled trials (RCTs) are relatively short term and, due to costs and available resources, have limited opportunity to be re-visited or extended. There is no guarantee that effects of treatments remain unchanged beyond the study. Here, we illustrate the feasibility, benefits and cost-effectiveness of enriching standard trial design with electronic follow up. We completed a 5-year electronic follow up of a RCT investigating the impact of probiotics on asthma and eczema in children born 2005–2007, with traditional fieldwork follow up to two years. Participants and trial outcomes were identified and analysed after five years using secure, routine, anonymised, person-based electronic health service databanks. At two years, we identified 93% of participants and compared fieldwork with electronic health records, highlighting areas of agreement and disagreement. Retention of children from lower socio-economic groups was improved, reducing volunteer bias. At 5 years we identified a reduced 82% of participants. These data allowed the trial’s first robust analysis of asthma endpoints. We found no indication that probiotic supplementation to pregnant mothers and infants protected against asthma or eczema at 5 years. Continued longer-term follow up is technically straightforward.

## Introduction

Follow up of trial participants on routine electronic health care databases offers great potential to maximise the economic efficiency of trials and allow access to a fuller range of study outcomes and potential adverse events^1–4^. Thus, the future use of electronic databases in clinical trials has been heralded as one of the major benefits to follow construction of nationwide electronic health records systems. To date, however, few studies have demonstrated this benefit, or formally assessed the relationship between traditional fieldwork data and electronic health care databases. Here, we report on the feasibility and efficiency of electronic follow up, and compare traditional trial fieldwork follow up with electronic follow up. We show new insights gained from outcomes electronically recorded 3 years after the end of the trial, and the congruence between fieldwork and electronic data.

## Methods

### The PROBAT Trial

The randomised, double-blind, placebo-controlled, parallel-group trial on which this follow-up is based was an assessment of the impact of a probiotic food supplement on prevention of the atopic conditions asthma, and eczema in young children. Healthy pregnant women were recruited between 2005 and 2007 to take supplement, or placebo, from 36 weeks’ gestation, and then administer it to their infants during their first 6 months of life. The supplement consisted of *lactobacilli* (*Lactobacillus salivarius* CUL61 NCIMB 30211 and *Lactobacillus paracasei* CUL08 NCIMB 30154) and *bifidobacteria* (*Bifidobacterium animalis subsp. lactis* CUL34 NCIMB 30172 and *Bifidobacterium bifidum* CUL20 NCIMB 30153) strains, administered at 1 × 10^10^ cfu per day^5,6^. The trial preparations were not marketed, but it remains a limitation that children or breastfeeding mothers in both arms may have taken commercial probiotics or live yoghurts during the extended follow up, however our primary hypotheses were based on elucidating the impact of very early exposure to specified organisms. Participants completed researcher-administered questionnaires, and attended clinics at regular intervals. Information was sought on health status, risk factors and symptoms of atopy, and adverse events^5^. Outcomes included associations between the probiotic supplement and reports of eczema and asthma at or before 2 years of age (see Tables S1, S2 in Supplementary Material).

### Linking trial and health service electronic data

The trial location allowed access to person-based routinely-collected electronic data collected by the National Health Service (NHS) and other public sector organisations in the UK, maintained at the Secure Anonymised Information Linkage (SAIL) databank at Swansea University Medical School^7,8^. Following ethical approval from South West Wales Research Ethics Committee on behalf of NHS Wales (project ref. 2004024), participants gave signed informed consent to 5-year follow up through medical records. This randomised, double blind, parallel group, placebo controlled trial is registered with Current Controlled Trials ISRCTN 26287422. The medical information accessed from General Practice (GP) and primary care records included all prescriptions and diagnoses. SAIL links a wide range of person-based datasets using robust irrevocable anonymisation, offering a valuable research resource whilst complying with UK data protection legislation^9^ and ensuring confidentiality. Following Information Governance Review Panel approval (reference number 0028), data were acquired from the databank in 2013 (after the last participant was 5 years old), and analysed 2014–15. All methods were performed in accordance with the terms and conditions of the Research Ethics Committee and Governance approvals (above).

Unique personal identifiers in PROBAT were trial study numbers linked to original recruitment records. Within SAIL, the identifier is known as an anonymised linkage field (ALF). The ALF is encrypted by a trusted third party [NHS Wales Informatics Service] and linked to clinical records within SAIL^10^. These two pieces of information were combined thus uploading the fieldwork into SAIL alongside health service data. In linking the data sets it was straightforward to retain blinding of study groups for all analysis, an advantage over open label study follow up. Confidentiality is maintained by the trusted third party anonymisation process, which includes reducing birthdates to week of birth and address to local area units of approximately 1500 people and if necessary aggregation of data across individuals. The area information allows calculation of Townsend indices of social deprivation. Data cannot be extracted from SAIL if patient anonymity can be compromised^10^, and published summary statistics must not identify a group of individuals less than 5 in number. Once all individual data is analysed in SAIL, odds ratios may need to be approximated (smoothed to a lower number of decimal points or significant figures) to ensure this. In our study, this was a very minor issue. On occasions we needed to restrict the odds ratio precision to 1 decimal point; these are indicated in the tables as ‘approximate’ statistics.

SAIL data were examined for NHS 5-byte version 2 Read codes^11^ relating to diagnoses of asthma and eczema, asthma related medicines, and antibacterial prescriptions. Asthma diagnosis in childhood is complex, and becomes more reliable as a child ages. As a primary outcome definition, rather than simply reported (Read coded) diagnoses at this young age (2–5 years); we accessed and included prescribed medication, specifically: more than one prescription of a beta2 agonist, or, a recorded diagnosis (Read code) of asthma plus at least one single prescription of a beta2 agonist. For sensitivity analysis, we also considered a range of 5 definitions, representing increased likelihood of a true positive diagnosis, classified according to British Thoracic Society guidelines^12,13^ (Tables S3, S4). Asthma definitions were run as Structured Query Language (SQL) queries based on lists of Read codes for (anonymised) trial participants in the databank. Eczema diagnosis was relatively straightforward, defined as presence of Read codes for diagnoses of infantile eczema, flexural eczema, infected eczema, atopic dermatitis/eczema, and eczema not otherwise specified.

A crucial factor is the extent of electronic data coverage. In the setting of this trial, GP practices are incentivised to accurately code certain medical conditions under the Quality and Outcomes Framework^14,15^, a process of financial reward based on points systems for certain clinical indicators, including long term monitoring of patients with asthma. Like many study resources, electronic records have missing information. However, unlike a designed study, one cannot easily identify missing cells. A gap in the electronic record can reflect failure of the record, or simply absence of any clinical event at that time. We defined ‘Good’ data coverage, and therefore successful follow up, according to an algorithm based on: the frequency of any event in the GP practice in the period being at least 10% of that measured from Jan 1^st^ to Dec 30^th^ 2009 (a period known to have good coverage); the absence of gaps in the individual’s or GP practice’s record of greater than 30 days. It is noted that the algorithm is *ad hoc*, but serves to identify GP practices that do not submit data regularly and individuals who have deregistered from their primary care practices, for example by moving away from the area.

### Statistical Analysis

For children (trial subjects) present with good data coverage at 2 and 5 years, trial outcomes and covariates were assessed using binary logistic regression. Any variables identified as statistically significant (5% level) using univariable analysis were investigated in multivariable logistic regression adjusted models, with a final model chosen by likelihood ratio tests (with a variable retained in the final model if the model showed a significant improvement in fit). To explore the sensitivity of the conclusions to issues of trial compliance and uncertainty in the definition of asthma we undertook ITT (intention to treat) and PP (*per protocol*) analyses for asthma and eczema at 5 years, in combination with all 5 reasonable asthma classifications. We defined good compliance as infants taking >70% of the treatment (30 or more treatments) by 6 weeks of age.

Comparison of electronic and field data was made using Cohen’s Kappa, with levels of agreement as defined by Landis *et al*.^16^. In all tables we report p-values without adjustment for multiple comparisons. This was due to the high degree of correlation between endpoints (for example the different, but nested, definitions of asthma) making formal adjustment difficult. However we note that without any adjustment, P-values should be treated with the usual caution, and interpreted within the context of the number of tests performed. All analyses were completed before treatment group allocation was un-blinded.

## Results

### Feasibility of electronic follow-up

**Figure 1:**
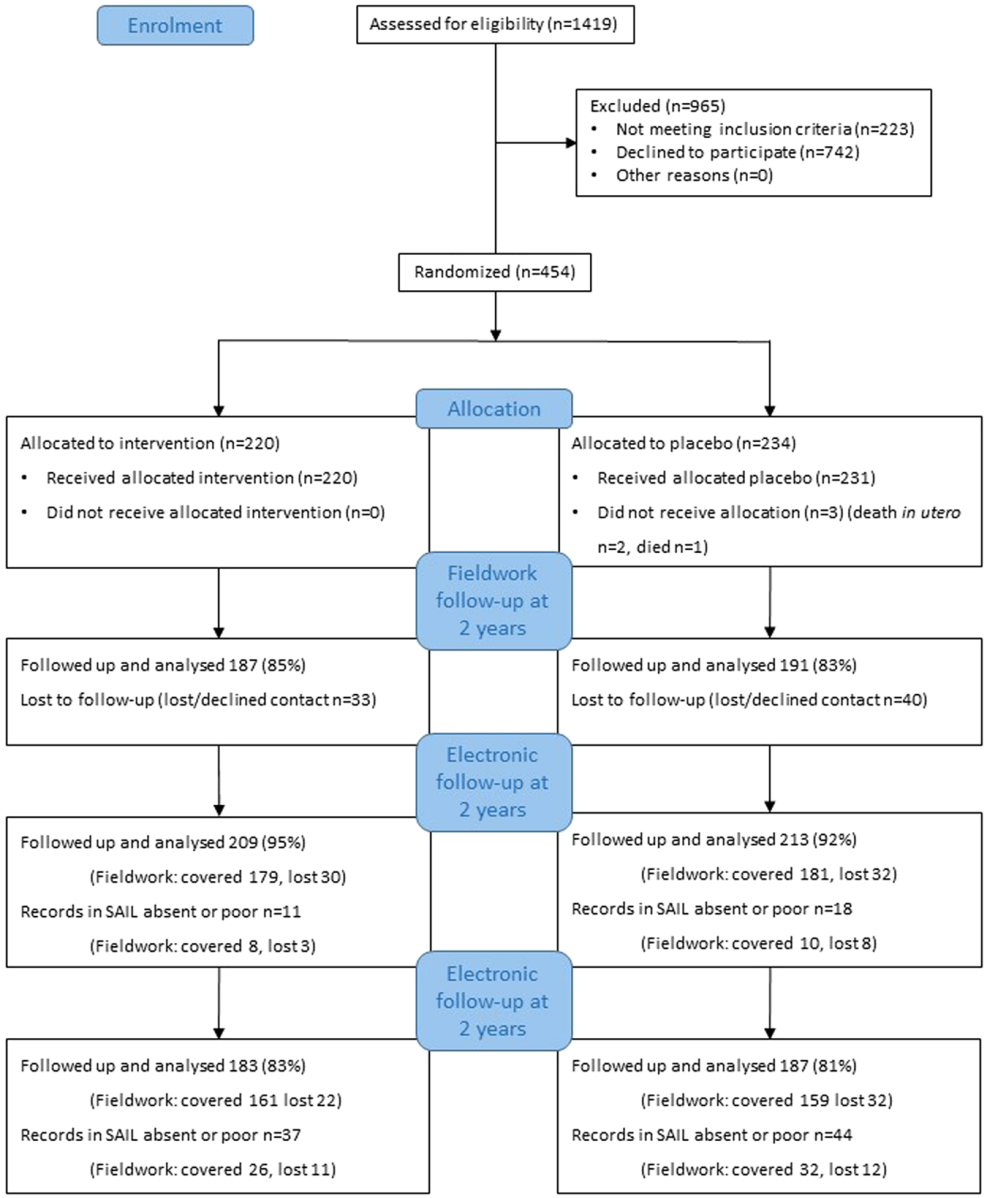
Trial Profile: the PROBAT trial with 5 year follow up. see refs^5,6,40^.

**Figure 2:**
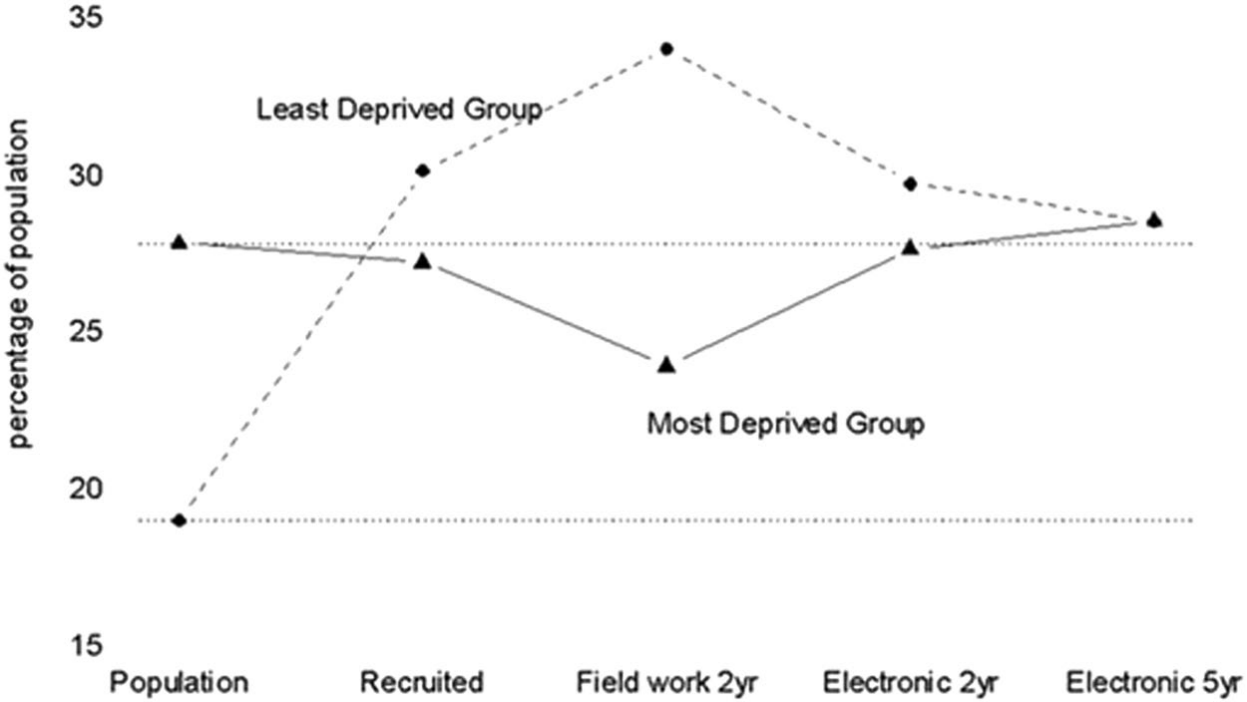
Trends in recruitment and retention of trial participants via fielder ork and electronic follow up. Our study was conducted in a population with a high percentage (28%. dotted horizontal line) of people in the lowest socio-economic fifth (defined by the Townsend Index), and low percentage in the highest socio-economic fifth (18%). Although recruitment to the trial from the most deprived group was excellent, this group was under-represented in field work follow-up at 2 years. The impact of this was explored elsewhere^3^. However, this loss was rectified in electronic records at both 2 years and 5 years (solid line). The trial over-recruited in the least deprived group; this discrepancy was exacerbated in field work, but improved by electronic follow up (dashed line). Deprivation (Townsend) fifths are based on geographical area of residence, using Lower Super Output Areas (LSOAs) defined by postcodes. This measure of material deprivation is calculated from rates of unemployment, vehicle ownership, home ownership, and overcrowding^45^.

435 of 452 (96%) trial participants were located in the SAIL databank. Good coverage was identified for 422 participants at 2 years (93%) and 370 participants at 5 years (82%). The electronically extended trial profile is illustrated in Fig. 1. Interestingly, loss-to-follow up of the most deprived (assessed by Townsend score) was less in electronic than traditional trial data. Figure 2 shows that although great efforts were initially made to start with a representative proportion of the lowest socio-economic fifth, this group was more likely to be lost to follow up in field work. In contrast, these participants were recovered in 2 year electronic follow up and retained at 5 year electronic follow up (see also Table S1 for demographic details).

### Comparison of electronic data with fieldwork at 2 years

For asthma outcomes, 318 participants were present in both electronic and fieldwork datasets. For eczema and antibacterial usage, the numbers were 321 and 408 respectively. All comparisons between electronic and field data showed ‘fair’ agreement (kappa values 0.26 to 0.54), but all also fell short of ‘substantial’ agreement (a kappa value >0.60) (Table S9).

The disagreements can be examined in more detail. 23 participants were classified as having asthma in the 2 year fieldwork. Of these, only 10 had a general practitioner diagnosis or had been prescribed >1 beta2 agonist prescription in the electronic records. Coincidentally, exactly 23 participants were also classified as asthmatic in the electronic data, with 10 of these not having been reported as such to the fieldworkers. Agreement for antibiotic exposure was little better than chance.

### Analysis of trial outcomes using electronic follow-up data at 2 and 5 years

Briefly, at 2 and 5 years, both ITT and PP analyses indicated a higher prevalence of asthma in the children in the probiotic arm in univariable and multivariable analyses. This difference was statistically significant (not adjusted for multiple comparisons) in PP analyses only, and in both sets of analyses the association at 5 years was less than that found at 2 years. Table 1 shows absolute risk differences for the treatment groups at 5 year electronic follow up. The number of participants remaining in the PP analyses was considerably reduced (Table 1), which was a consequence of a strict definition of compliance (>30 treatments), providing a clear contrast with the ITT groups. Approximate (to comply with SAIL data release policy^10^) and exact unadjusted odds ratios were: 2 yr PP OR ~5 (1.6 to 15.0); 5 yr PP OR = 2.18 (1.06 to 4.50); 2 year ITT OR = 1.43 (0.74 to 2.75); 5 yr ITT OR = 1.13 (0.69 to 1.84).

**Table 1:** Main asthma and eczema outcomes at 5 years.

	n(%) with condition exposed to probiotics	n(%) with condition not exposed	Absolute risk difference (95% CI) Between exposed and not exposed	NNH
Asthma at 5 years *ITT analysis*	43/183 (23.5%)	40/187 (21.4%)	2.1% (−6.4–10.6%)^‡^	48
Asthma at 5 years *PP* analysis	26/84 (31.0%)	15/88 (17.0%)	13.9% (1.3–7.9%)^‡^ p < 0.05	8
				NNT
Eczema 5 years *ITT analysis*	61/183 (33.3%)	65/187 (34.8%)	1.4% (−8.2 to 11.1%)^§^	71
Eczema 5 years *PP* analysis	29/84 (34.5%)	33/88 (37.5%)	3.0% (−11.4 to 17.3%)^§^	34

Asthma primary definition based on prescription of more than one prescription of a beta2 agonist, or, a recorded diagnosis of asthma plus at least one single prescription of a beta2 agonist. ^‡^Favours placebo. ^§^Favours probiotics. NNH = number needed to harm, NNT = number needed to treat. Full details are in Supplementary Tables 5–8 and, for 2 year follow up, in ref.^6^, Table 5.

We next explored multivariable analysis, adjusting for background variables. In PP analysis at 2 years, no other demographic variables were associated with asthma. In PP analysis at 5 years, the probiotic arm was retained as significant in the final model, after adjustment for significant risk factors: smoking and paternal history of asthma. The odds ratio increased to 3.33 (1.47 to 7.14). Sensitivity of results to the definition of asthma, and compliance is given in full in Table S10. We found, after adjustment for any significant background variable, that the estimated odds ratio for the effect of the probiotic on asthma ranged between a high of 5.0 (asthma definition 2 at 2 years, PP analysis, 95% CI (1.5, 15) p = 0.01) to a low of 0.41 (asthma definition 3 at 2 years, ITT analysis, 95% CI (0.14, 1.2) p = 0.1). One odds ratio was exactly 1.0; 9/17 showed an odds ratio greater than one, and 7/17 showed an odds ratio below one. Thus there was no consistent evidence for a probiotic effect. Significant background risk factors included family history of allergy, absence of breastfeeding and smoking in the household (S10).

In all treatment group analyses, odds ratios for eczema were less than 1, indicating a trend for lower eczema in the probiotic arm, but no comparisons reached statistical significance at the 5% level. Demographic risk factors associated with eczema included attendance at daycare, keeping caged birds or rodents as pets, and paternal reported allergy. Full details are available in Tables 5–8 and sensitivity to compliance table in S10.

## Discussion

The benefit of electronic record linkage to long term trial follow was shown by the West of Scotland Coronary Prevention Study^1,17^, which was instrumental in expanding statin use for cardiovascular disease prevention. 10 years after the end of the trial, continued risk reduction was demonstrated along with no long-term safety concerns (see also the follow-up of the ISIS-2 trial via hospital records^18^). However, these approaches are still neither routine practice nor requirements of funding bodies. Using keywords ‘electronic health record’, ‘data linkage’, ‘clinical trial’, ‘follow-up’, ‘secondary use’, ‘comparative study’, in PubMed and SCOPUS (21/08/17) we found no other studies comparing findings from electronic health services’ databases with traditional fieldwork. This despite great concerns over study replication, a burgeoning literature proposing the use of electronic medical records in clinical research^19–23^ and projects such as The Electronic Healthcare Records for Clinical Research^24^. Here we show electronic follow up of an RCT, both at 2 years (within the trial period) and 5 years (beyond available resources in the traditional trial framework). We also introduce a further level of linkage: pregnant women were recruited to the trial, but follow up was extended to the more relevant population; children identified in the health records.

The proportion of recruits withdrawing from trials steadily increased between 1955 and 2000^25^, prompting the US Food and Drug Administration to insist on measures to minimise missing data^26^. This is not simply due to loss of power, but also the consequent bias if loss is non-random^27^. Where participants are lost from the socioeconomic group where disease prevalence is highest, usually the most deprived, power is further reduced^6^. Indeed, in our trial the lowest socio-economic group was less represented, but when electronic records were used, volunteer selection bias decreased. We note, given our loss to follow up is 18% at 5 years, that additional biases related to inclusion in databases may still be present. However, this is within the 80–90% follow up rates that are generally considered acceptable^28^.

Electronic medical records are increasingly used in observational studies to detect safety issues, such as adverse drug events^29^ and prescription errors^30^. SAIL has been used as a cost-effective resource for trial recruitment^31^, and evaluation of complementary and alternative medicines^32^. In SAIL cohort studies (ankylosing spondylitis^33^, psychotic disorders^34^), 80% of patients were located within the electronic records, comparable figures to our study at 5 years.

The level of agreement between data sources is difficult to interpret, especially where fieldwork involves reliance on carer recall. Neither fieldwork nor the electronic record can be considered the gold standard. Some endpoints are better suited to traditional fieldwork, and others to electronic health records. Fieldwork data are vulnerable to misunderstandings of questionnaires, perceptions or definitions of illness. Although prescription recording is accurate, the prescription itself may not be redeemed or used by the carer (see Gadkari *et al*.)^35^. Asthma has been identified as a condition for which compliance with primary care prescription is low^36^. Here, we propose the asthma endpoints are generally more reliable in the electronic follow up, due to the older age of the children and also the risk of bias or unreliability in carer-reported outcomes, where a range of concerns might be designated ‘asthma’. This might be reflected in the high proportion of children with fieldwork-reported asthma, yet no evidence of any prescription of asthma medicines. Antibiotic usage is likely a case where the more objective electronic data is preferred, due to difficulty in recalling medicine up to 12 months ago in the fieldwork, or misunderstandings over the term. On the other hand, electronic data may also be compromised by in-hospital prescription not recorded in the GP data. In contrast, certain outcomes such as atopic eczema cannot easily be reproduced in the electronic follow up, as the link to prescription medicines is less clear.

### Impact of probiotics on Clinical Outcomes

The electronic data at 2 years were in agreement with the original trial, and other literature, in failing to find a preventive role for probiotics on asthma outcomes^37,38^. We identified a possible increased asthma prevalence^39,40^, though this should be interpreted with caution as several endpoints were analysed in sensitivity analyses. Our study is the first to address longer term electronic follow up of infants exposed *in utero* and suggests that even if there are any associations, they tend to diminish over time.

In the original trial, at 2 years, the probiotic supplementation appeared to confer some benefits by reducing atopic eczema (not ‘any eczema’)^6,41^. This diagnosis required skin prick testing and was therefore unsuited to electronic follow up. Meta-analysis^42^ of studies investigating probiotics’ impact on more widely defined eczema, as investigated here, suggests a possible preventative effect. This was not supported by any statistically significant findings in our study. We note that our estimated effect sizes were consistent with published pooled relative risks of between 0.62 and 0.78, however we also found that the odds ratio in our study moved closer to 1 between 2 and 5 years follow up.

### Strengths and Limitations

The advantages of electronic follow up include minimisation of reporter, recall, social desirability response bias or mis-understandings in questionnaires. However, constraints or missing values in health records may include: absence of medicines prescribed in secondary care (soon to be available in SAIL) or privately purchased; absence of free text clinical notes; prescriptions given ‘just in case’; non-adherence to prescription regimens; quality of coding; absence of confounding variables (although many demographic variables can be recorded, trials will often require additional information). We acknowledge that prescriptions of asthma medicines at 2 and 5 years may not indicate a formal diagnosis of asthma; rather, they are functional measures of primary care practitioners’ perceptions of respiratory illness. Extension to beyond 7 years is straightforward (with ethical approval) and would offer a definitive asthma endpoint and further illustration of the advantages of the enriched trial.

Electronic follow up is likely to be cost-effective. Health informaticians can match and extract data quickly, here, some 6 months of analyst’s time, plus statistical re-analysis, research governance approvals, checking against data held by the Office of National Statistics (ONS), and checking with fieldwork data. We estimate the cost to be $80k ($57k in human resources), ~$200 per participant, which compares favourably with a mean of $24,727 per participant for patient retention in phase 3 pharmaceutical trials^43^. Although full cost-benefit analysis would require quantification of data quality, unlike traditional methods, the cost of follow-up using routine data is potentially relatively small and crucially does not increase with the number of participants. However, an infrastructure must be in place and meet exacting safeguarding standards^44^. SAIL, for example, is a large project supported by data scientists and significant computing hardware. This approach is still cost-effective over even a small number of trials. Given the cost of a suite of trials (our experience with PROBAT was approximately $2000 per participant) it is imperative that as much information, over a period as long as possible, is utilised. The potential bias introduced whenever there is loss of data within the trial framework must always be considered, however we suggest that the benefits of updating and re-testing trial hypotheses are such that trial funders should routinely recognise and support infrastructure for long-term follow up, using centres offering comprehensive electronic follow up^7,8^.

### Key Points

- Electronic follow-up of a clinical trial involving UK participants using the SAIL databank was feasible and effective, with a high proportion (96%) of participants identified with good data coverage.
- At 2 years of age retention was greater in electronic records, and with reduced attrition bias in lower socio-economic status groups.
- 5 years, retention was still high and free of bias in socio-economic status. Future extension of the trial is straightforward within this framework.
- 5 year electronic follow up did not find support for an effect of early use of probiotics on childhood eczema or asthma.
- The benefits relating to cost-effective, long term monitoring of complex interventions have implications for future clinical trial design.

## Electronic supplementary material

Supplementary Material
