# Supplementary material for: Long term extension of a randomised controlled trial of probiotics using electronic health records
Source: Sci Rep. 2018 May 16;8:7668. doi: 10.1038/s41598-018-25954-z (PMC5955897; doi:10.1038/s41598-018-25954-z)
Supplement: Supplementary file 1 — Supplementary Material [file 41598_2018_25954_MOESM1_ESM.docx]

**Supplementary Material**

**Long term extension of a randomised controlled trial of probiotics using electronic health records**

Gareth Davies MSc^1†^ Sue Jordan PhD^2*†^, Caroline Brooks BSc^1^, Daniel Thayer BSc^1^, Melanie Storey MSc^2^, Gareth Morgan MD^3,4^ Stephen Allen MD^5^, Iveta Garaiova PhD^6^, Sue Plummer PhD^6^, Mike Gravenor DPhil^1†^

^1^Swansea University Medical School, Singleton Park, Swansea, UK

^2^Department of Nursing, The College of Human and Health Sciences, Swansea University, Singleton Park, Swansea, UK

^3^The Children's Trust, The HCA Portland Hospital for Women and Children, London UK.

^4^Great Ormond Street Hospital for Children, London, UK

^5^Liverpool School of Tropical Medicine, Pembroke Place, Liverpool, UK

^6^Research Department, Cultech Limited, Baglan Industrial Park, Port Talbot, UK

* corresponding author. The College of Human and Health Sciences, Swansea University, Singleton Park, Swansea, Wales, UK. S.E.Jordan@swansea.ac.uk

^†^These authors jointly led this work

**Table S1. Data collection in the PROBAT trial**

| 1. Participants were asked to respond to questionnaires regarding compliance with trial interventions, putative risk factors for atopy, signs and symptoms of atopic conditions, adverse events (defined as any untoward medical occurrence in a patient or clinical investigational subject administered a pharmaceutical product and which does not necessarily have to have a causal relationship with this treatment.) and infant’s health at:   - 36 weeks of pregnancy (recruitment) - When infant was 6, 12, 18, 24, 52 and 104 weeks of age - Clinic visits scheduled at 6 months and 2 years.   Questionnaires were administered face to face at research clinics or in participants’ homes or over the telephone.  2. Participants were asked for consent to access:   - maternity records on pregnancy and childbirth - children’s medical records   3. The following biological samples were requested:   - maternal blood at 36 weeks, taken with routine ante-natal blood samples, if possible. - the placenta after delivery (placentas are normally discarded after delivery). - blood from the umbilical cord (after separation), after the birth of the infant. - blood samples (5 mls taken from the back of the hand or forearm after using an anaesthetic cream) at 6 months. - skin-prick tests for common allergens at age 6 months and 2 years. This involves putting common allergens on the skin in drops of fluid, scratching the skin very gently and observing local skin reactions. - breast milk from breastfeeding mothers at 2 & 6 weeks after delivery (home visit, subsample only). - stool samples from nappies at birth, 2, 6, 12, 18 and 24 weeks. (Soiled nappy placed in a sealed envelope.)   Sample collection necessitated additional contacts, immediately after birth and 2 weeks post partum. |
| --- |

**Table S2. Two year questionnaire section on reported diagnoses**

| Since the last assessment at age 1 year, has your child had any of the following?  Codes for answers:  1 = Yes and diagnosed by a health professional  2 = No  3 = Yes but NOT diagnosed by a health professional  4 = Don’t know  9 = Missing data  If participant answers “No” – go down the list to check |
| --- |
| - Any skin problem (other than an infection)? |
| - - Dry skin only |
| - - **Eczema** |
| - Hives, nettle-rash or urticaria (episodes of red itchy wheals/ blisters) |
| - - **Skin allergy or contact dermatitis; If Yes: give details**: |
| ……………………………………………………………………. |
| - - Psoriasis |
| - - Other skin problem: details ....……………………………....... |
|  |
| - Any respiratory / breathing problem? |
| - - **asthma (if unclear ask if asthma medicines or inhalers used)** |
| - any other breathing / respiratory problem; details: |
|  |
| - **Hay fever / allergic rhinitis (seasonal episodes of sneezing, runny and itchy nose and watery and itchy eyes)** |
| Allergies to: |
| - Cow milk |
| - Egg |
| - Cat |
| - Pollen |
| - Dust |
| - Insect bites or stings |
| - Something else, details ……………………………………………........ |
|  |
| - Does your child receive regular treatment for any ‘allergic’ problems? details: |
| **In the last year**, for how many DAYS has your child received:  *(000 =none; 888 = don’t know, 999 =missing data)*  *Prompts: Has your child ever had …. If so, on how many occasions …*  *Or, how often …. Every day, every week … how many times each week?*  (multiply number of times each week by 52)   - **Antibiotic; details**: .................................................................................... - Steroids; details: ...................................................................................... - Antihistamines - Creams or ointments for eczema (details.................................................) - Paracetamol - Other medicines; details: ......................................................................... |

**Table S3. Prescriptions indicative of asthma**

| **Asthma stage** | **Diagnostic Criteria** |
| --- | --- |
| 1 | Any prescription of a short acting beta 2 agonist  (This may be a therapeutic trial indicating respiratory symptoms) |
| 2 | (Equivalent to BTS step 1)  More than one prescription of a short acting beta 2 agonist  OR  One prescription of a short acting beta 2 agonist plus a GP diagnosis of asthma |
| 3 | (Equivalent to BTS step 2)  Any prescription of an inhaled corticosteroid  OR  Any prescription of a leukotriene receptor antagonist without a corticosteroid |
| 4 | (Equivalent to BTS step 3)  Any prescription of an inhaled corticosteroid plus either a leukotriene receptor antagonist* or a long acting beta 2 agonist (alternative add-on therapy) |
| 5 | Any prescription of an oral corticosteroid plus a concurrent asthma diagnosis at time of prescription (within 14 days)  OR  A past history of asthma medication stages 2 to 4 above |
| **recommended ‘add-on’ for <5s in BTS/SIGN guidelines 2012* | |

**Table S4. Algorithm for asthma diagnostic criteria for coding into Semi-English Query Language (SQL)**


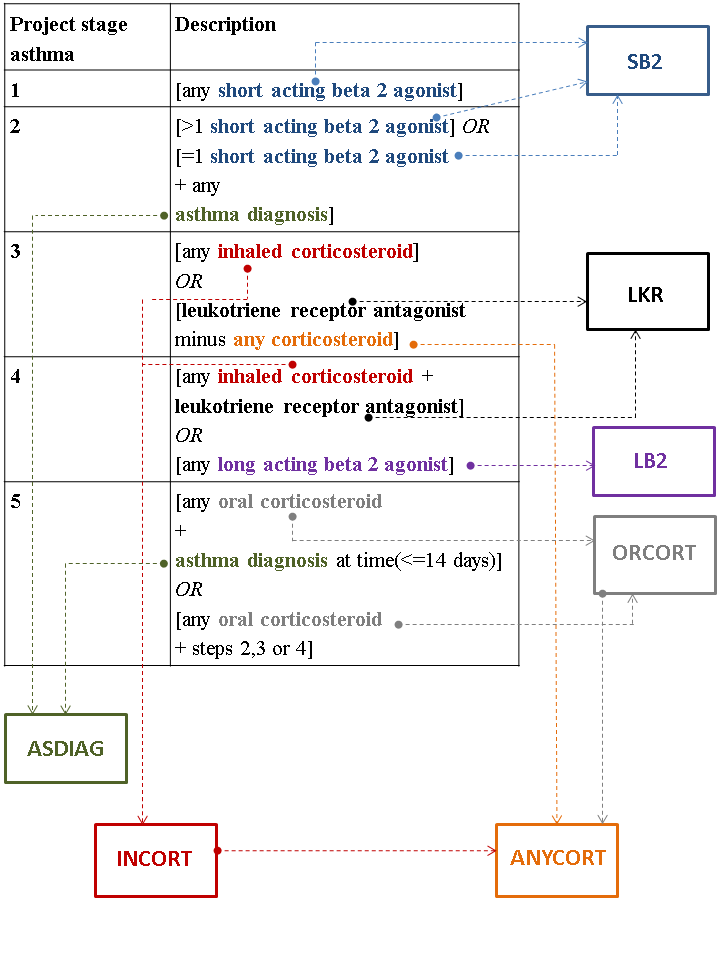


**Note to figure 1:** The five asthma diagnosis stages are broken down into their constituent elements and then defined as NHS Read code lists representing the relevant medicines and diagnostic terms. Membership of the stage can be determined by examining the Read code events and event date for each participant.

**Key to figure 1:**

SB2 - Read code list to define short acting beta 2 agonists

ASDIAG - Read code list to define diagnosis of asthma by GP

INCORT - Read code list to define inhaled corticosteroids

LKR - Read code list to define leukotriene receptor antagonists

LB2 - Read code list to define long acting beta 2 agonists

ORCORT - Read code list to define oral corticosteroids

ANYCORT - Read code list to define corticosteroids in general (=ORCORT+INCORT)**Table S5. Prevalence of eczema and asthma and antibacterial prescriptions at 2 and 5 years**

| **Variable** | **Numbers** | | | | | |
| --- | --- | --- | --- | --- | --- | --- |
|  | **Unadjusted raw data** | | **Good electronic coverage, ITT analysis** | | **Good electronic coverage, PP analysis** | |
|  | **2yrs – total, probiotic, placebo** | **5yrs – total, probiotic, placebo** | **2yrs – total, probiotic, placebo** | **5yrs – total, probiotic, placebo** | **2yrs – total probiotic, placebo** | **5yrs – total probiotic, placebo** |
|  | | | | | | |
| Population | 435 (100%),  212,223 | 435 (100%),  212,223 | 422 (100%),  209,213 | 370 (100%),  183,187 | 195 (100%),  95,100 | 172 (100%),  84,88 |
|  | | | | | | |
| Asthma stage 1 | 70 (16.1%),  33,37 | 136 (31.3%),  62,74 | 69 (16.4%),  32,37 | 116 (31.4%),  54,62 | 35 (17.9%),  20,15 | 59 (34.3%),  31,28 |
| Asthma stage 2 | 41 (9.4%),  24,17 | 95 (21.8%),  48,47 | 40 (9.5%),  23,17 | 83 (22.4%),  43,40 | 20 (10.3%),  >15,<5 | 41 (23.8%),  26,15 |
| Asthma stage 3 | 17 (3.9%),  5,12 | 52 (12.0%),  24,28 | 17 (4.0%),  5,12 | 45 (12.2%),  22,23 | 5 (2.6%), | 18 (10.5%),  12,6 |
| Asthma stage 4 | <5 | 10 (2.3%), | <5 | 9 (2.4%), | <5 | 5 (2.9%), |
| Asthma stage 5 | 7 (1.6%) | 26 (6.0%),  13,13 | 7 (1.7%) | 23 (6.2%),  12,11 | <5 | 10 (5.8%), |
|  | | | | | | |
| Eczema | 100 (23.0%),  44,56 | 144 (33.1%),  70,74 | 100 (23.7%),  44,56 | 126 (34.1%),  61,65 | 50 (25.6%),  21,29 | 62 (36.0%),  29,33 |
|  | | | | | | |
| Antibacterials | 149 (34.3%),  73,76 | 266 (61.1%),  141,125 | 146 (34.6%),  72,74 | 235 (63.5%),  124,111 | 75 (38.5%),  39,36 | 110 (64.0%),  59,51 |

Notes: We are unable to disclose numbers 1-4. ITT analysis = any compliance, PP analysis = compliance >70%

Asthma stages are outlined in Table S3

**Table S6. Unadjusted univariate analysis at 2yrs - Asthma & Eczema, Intention to treat & *Per Protocol***

|  | | **AsthmaITT** | | | | **Asthma PP** | | | | **Eczema ITT** | | | | **Eczema PP** | | | |
| --- | --- | --- | --- | --- | --- | --- | --- | --- | --- | --- | --- | --- | --- | --- | --- | --- | --- |
| **Covariate** | **Status** | **Total**  **N** | **N of**  **events** | **p-**  **val** | **Odds Ratio**  **(95% CI)** | **Total**  **N** | **N of**  **events** | **p-**  **val** | **Odds Ratio**  **(95% CI)** | **Total**  **N** | **N of**  **events** | **p-**  **val** | **Odds Ratio**  **(95% CI)** | **Total**  **N** | **N of**  **events** | **p-**  **val** | **Odds Ratio**  **(95% CI)** |
| **Treatment group** | Placebo | 213 | 17 | 0.29 | 1.43  (0.74 to 2.75) | **100**  **95** | **<5**  **16-19** | **0.01** | **~5**  **(~1.6 to ~15)** | 213  209 | 56  44 | 0.21 | 0.75  (0.48 to 1.17) | 100  95 | 29  21 | 0.27 | 0.69  (0.36 to 1.33) |
|  | Probiotic | 209 | 23 |  |  |  |  |  |  |  |  |  |  |  |  |  |  |
| Delivery method | Vaginal | 128 | 8 | 0.16 | 1.80  (0.80 to 4.02) | 60  135 | 6  14 | 0.94 | 1.04  (0.38 to 2.86) | 128  290 | 32  67 | 0.67 | 0.90  (0.56 to 1.46) | 60  135 | 16  34 | 0.83 | 0.93  (0.46 to 1.85) |
|  | C section | 290 | 31 |  |  |  |  |  |  |  |  |  |  |  |  |  |  |
| **Any breastfeeding** | **No** | **127** | **18** | **<0.01** | **0.25**  **(0.10 to 0.61)** | 67  95 | 8  7 | 0.33 | 0.59  (0.20 to 1.70) | 127  179 | 30  49 | 0.46 | 1.22  (0.72 to 2.06) | 67  95 | 19  26 | 0.89 | 0.95  (0.47 to 1.91) |
|  | **Yes** | **179** | **7** |  |  |  |  |  |  |  |  |  |  |  |  |  |  |
| **Any daycare attendance** | No | 256 | 25 | 0.49 | 0.78  (0.38 to 1.59) | 112  83 | 11  9 | 0.82 | 1.12  (0.44 to 2.83) | **256**  **155** | **52**  **46** | **0.03** | **1.66**  **(1.05 to 2.62)** | 112  83 | 24  26 | 0.12 | 1.67  (0.88 to 3.19) |
|  | Yes | 155 | 12 |  |  |  |  |  |  |  |  |  |  |  |  |  |  |
| Any siblings | No | 228 | 16 | 0.06 | 1.87  (0.96 to 3.63) | 107  88 | 8  12 | 0.16 | 1.95  (0.76 to 5.02) | 228  194 | 59  41 | 0.25 | 0.77  (0.49 to 1.21) | 107  88 | 28  22 | 0.85 | 0.94  (0.49 to 1.80) |
|  | Yes | 194 | 24 |  |  |  |  |  |  |  |  |  |  |  |  |  |  |
| Mother smoked | No | 353 | 31 | 0.27 | 1.56  (0.71 to 3.44) | 171  24 | 16-19  <5 | 0.74 | ~0.8  (~0.2 to ~4) | 353  69 | 85  15 | 0.68 | 0.88  (0.47 to 1.63) | 171  24 | 41  9 | 0.16 | 1.90  (0.78 to 4.67) |
|  | Yes | 69 | 9 |  |  |  |  |  |  |  |  |  |  |  |  |  |  |
| Household smoked | No | 249 | 20 | 0.23 | 1.50  (0.78 to 2.87) | 119  76 | 12  8 | 0.92 | 1.05  (0.41 to 2.70) | 249  173 | 59  41 | 1.00 | 1.00  (0.63 to 1.58) | 119  76 | 27  23 | 0.24 | 1.48  (0.77 to 2.83) |
|  | Yes | 173 | 20 |  |  |  |  |  |  |  |  |  |  |  |  |  |  |
| Any pets | No | 210 | 19 | 0.76 | 1.11  (0.58 to 2.12) | 94  101 | 9  11 | 0.76 | 1.15  (0.46 to 2.92) | 210  212 | 49  51 | 0.86 | 1.04  (0.66 to 1.63) | 94  101 | 23  27 | 0.72 | 1.13  (0.59 to 2.15) |
|  | Yes | 212 | 21 |  |  |  |  |  |  |  |  |  |  |  |  |  |  |
| Cats | No | 324 | 29 | 0.50 | 1.29  (0.62 to 2.68) | 152  43 | 13  7 | 0.15 | 2.08  (0.77 to 5.59) | 324  98 | 79  21 | 0.55 | 0.85  (0.49 to 1.46) | 152  43 | 38  12 | 0.70 | 1.16  (0.54 to 2.49) |
|  | Yes | 98 | 11 |  |  |  |  |  |  |  |  |  |  |  |  |  |  |
| Dogs | No | 305 | 28 | 0.74 | 1.13  (0.55 to 2.31) | 136  59 | 14  6 | 0.98 | 0.99  (0.36 to 2.71) | 305  117 | 72  28 | 0.94 | 1.02  (0.62 to 1.68) | 136  59 | 34  16 | 0.76 | 1.12  (0.56 to 2.23) |
|  | Yes | 117 | 12 |  |  |  |  |  |  |  |  |  |  |  |  |  |  |
| Rabbits | No | 404 | 36-39 | 0.57 | ~0.6  (~0.1 to ~4) | 184  11 | 16-19  <5 | 0.90 | ~0.9  (~0.1 to ~7) | 404  18 | 96-99  <5 | 0.88 | ~0.9  (~0.3 to ~3) | 184  11 | 46-49  <5 | 0.90 | ~1.1  (~0.3 to ~4) |
|  | Yes | 18 | <5 |  |  |  |  |  |  |  |  |  |  |  |  |  |  |
| Rodents | **No** | 399 | 36-39 | 0.19 | ~2  (~0.7 to ~7) | 185  10 | 20  0 | *NA* | *NA* | 399  23 | 92  8 | 0.20 | 1.78  (0.73 to 4.33) | 185  10 | 45  5 | 0.08 | 3.11  (0.86 to 11.24) |
|  | **Yes** | 23 | <5 |  |  |  |  |  |  |  |  |  |  |  |  |  |  |
| **Birds** | No | 410 | 40 | *NA* | *NA* | 188  7 | 20  0 | *NA* | *NA* | **410**  **12** | **94**  **6** | **0.04** | **3.36**  **(1.06 to 10.6)** | **188**  **7** | **45**  **5** | **0.02** | **7.94**  **(1.49 to 42.36)** |
|  | Yes | 12 | 0 |  |  |  |  |  |  |  |  |  |  |  |  |  |  |
| **In least deprived fifth** | **No** | **297** | **36-39** | **0.01** | **~0.2**  **(~0.1 to ~0.7)** | 131  64 | 16-19  <5 | 0.09 | ~0.3  (~0.1 to ~1.2) | 297  125 | 78  22 | 0.06 | 0.60  (0.35 to 1.02) | 131  64 | 38  12 | 0.13 | 0.56  (0.27 to 1.17) |
|  | **Yes** | **125** | **<5** |  |  |  |  |  |  |  |  |  |  |  |  |  |  |
| **In most deprived fifth** | **No** | **306** | **21** | **<0.01** | **2.66**  **(1.37 to 5.15)** | 148  47 | 13  7 | 0.23 | 1.82  (0.68 to 4.86) | 306  116 | 77  23 | 0.25 | 0.74  (0.44 to 1.24) | 148  47 | 42  8 | 0.12 | 0.52  (0.22 to 1.20) |
|  | **Yes** | **116** | **19** |  |  |  |  |  |  |  |  |  |  |  |  |  |  |
| Household damp | No | 311 | 31 | 0.64 | 0.83  (0.38 to 1.80) | 143  51 | 15  5 | 0.89 | 0.93  (0.32 to 2.70) | 311  107 | 74  25 | 0.93 | 0.98  (0.58 to 1.64) | 143  51 | 38  12 | 0.67 | 0.85  (0.40 to 1.79) |
|  | Yes | 107 | 9 |  |  |  |  |  |  |  |  |  |  |  |  |  |  |
| Household mould | No | 354 | 34 | 0.98 | 0.99  (0.40 to 2.47) | 158  37 | 16-19  <5 | 0.90 | ~1.1  (~0.3 to ~3) | 354  63 | 83  16 | 0.74 | 1.11  (0.60 to 2.06) | 158  37 | 40  10 | 0.83 | 1.09  (0.49 to 2.45) |
|  | Yes | 63 | 6 |  |  |  |  |  |  |  |  |  |  |  |  |  |  |
| High risk doctor diagnosed | No | 57 | 7 | 0.44 | 0.71  (0.30 to 1.69) | 25  170 | <5  16-19 | 0.69 | ~1.4  (~0.3 to ~6) | 57  365 | 11  89 | 0.40 | 1.35  (0.67 to 2.72) | 25  170 | 5  45 | 0.49 | 1.44  (0.51 to 4.06) |
|  | Yes | 365 | 33 |  |  |  |  |  |  |  |  |  |  |  |  |  |  |
| High risk self diagnosed | No | 35 | <5 | 0.85 | ~1.1  (~0.3 to ~4) | 16  179 | 0  20 | *NA* | *NA* | 35  387 | 5  95 | 0.18 | 1.95  (0.74 to 5.17) | 16  179 | <5  46-49 | 0.95 | ~1.1  (~0.3 to ~3) |
|  | Yes | 387 | 36-39 |  |  |  |  |  |  |  |  |  |  |  |  |  |  |
| Mother allergy self reported | No | 107 | 8 | 0.42 | 1.40  (0.62 to 3.14) | 46  149 | <5  16-19 | 0.15 | 3.02  (0.67 to 13.55) | 107  315 | 26  74 | 0.87 | 0.96  (0.57 to 1.60) | 46  149 | 13  37 | 0.64 | 0.84  (0.40 to 1.76) |
|  | Yes | 315 | 32 |  |  |  |  |  |  |  |  |  |  |  |  |  |  |
| Father allergy self reported | No | 43 | <5 | 0.56 | ~1.4  (~0.4 to ~5) | 26  165 | <5  15-18 | 0.77 | ~0.8  (~0.2 to ~3) | 43  370 | 8  89 | 0.43 | 1.39  (0.62 to 3.10) | 26  165 | 5  44 | 0.42 | 1.53  (0.54 to 4.30) |
|  | Yes | 370 | 35-38 |  |  |  |  |  |  |  |  |  |  |  |  |  |  |
| Mother allergy doctor diagnosed | No | 161 | 16 | 0.80 | 0.92  (0.47 to 1.79) | 68  127 | 6  14 | 0.63 | 1.28  (0.47 to 3.50) | 161  261 | 38  62 | 0.97 | 1.01  (0.64 to 1.60) | 68  127 | 18  32 | 0.85 | 0.94  (0.48 to 1.83) |
|  | Yes | 261 | 24 |  |  |  |  |  |  |  |  |  |  |  |  |  |  |
| **Father allergy doctor diagnosed** | No | 215 | 23 | 0.37 | 0.73  (0.38 to 1.43) | 100  91 | 11  8 | 0.61 | 0.78  (0.30 to 2.03) | **215**  **198** | **37**  **60** | **<0.01** | **2.09**  **(1.31 to 3.33)** | **100**  **91** | **18**  **31** | **0.01** | **2.35**  **(1.21 to 4.60)** |
|  | Yes | 198 | 16 |  |  |  |  |  |  |  |  |  |  |  |  |  |  |
| *Note: Small numbers have been masked to maintain anonymity. Independent variables showing significance to the 5% level are highlighted in bold typeface with grey background.* | | | | | | | | | | | | | | | | | |

**Table S7. Unadjusted univariate analysis at 5yrs - Asthma & Eczema, Intention to treat & *Per Protocol***

|  | | **AsthmaITT** | | | | **Asthma PP** | | | | **Eczema ITT** | | | | **Eczema PP** | | | |
| --- | --- | --- | --- | --- | --- | --- | --- | --- | --- | --- | --- | --- | --- | --- | --- | --- | --- |
| **Covariate** | **Status** | **Total**  **N** | **N of**  **events** | **p-**  **val** | **Odds Ratio**  **(95% CI)** | **Total**  **N** | **N of**  **events** | **p-**  **val** | **Odds Ratio**  **(95% CI)** | **Total**  **N** | **N of**  **events** | **p-**  **val** | **Odds Ratio**  **(95% CI)** | **Total**  **N** | **N of**  **events** | **p-**  **val** | **Odds Ratio**  **(95% CI)** |
| **Treatment group** | Placebo | 187 | 40 | 0.63 | 1.13  (0.69 to 1.84) | **88**  **84** | **15**  **26** | **0.03** | **2.18**  **(1.06 to 4.50)** | 187  183 | 65  61 | 0.77 | 0.94  (0.61 to 1.44) | 88  84 | 33  29 | 0.68 | 0.88  (0.47 to 1.64) |
|  | Probiotic | 183 | 43 |  |  |  |  |  |  |  |  |  |  |  |  |  |  |
| Delivery method | Vaginal | 117 | 22 | 0.30 | 1.33  (0.77 to 2.31) | 56  116 | 11  30 | 0.37 | 1.43  (0.65 to 3.11) | 117  250 | 38  87 | 0.66 | 1.11  (0.70 to 1.77) | 56  116 | 18  44 | 0.46 | 1.29  (0.66 to 2.53) |
|  | C section | 250 | 59 |  |  |  |  |  |  |  |  |  |  |  |  |  |  |
| **Any breastfeeding** | No | **114** | **33** | **0.02** | **0.51**  **(0.29 to 0.91)** | 61  86 | 20  17 | 0.08 | 0.51  (0.24 to 1.07) | 114  162 | 42  62 | 0.81 | 1.06  (0.65 to 1.74) | 61  86 | 22  36 | 0.48 | 1.28  (0.65 to 2.51) |
|  | Yes | **162** | **28** |  |  |  |  |  |  |  |  |  |  |  |  |  |  |
| Any daycare attendance | No | 218 | 50 | 0.53 | 0.85  (0.51 to 1.42) | 94  78 | 24  17 | 0.57 | 0.81  (0.40 to 1.65) | 218  144 | 68  58 | 0.08 | 1.49  (0.96 to 2.31) | 94  78 | 30  32 | 0.22 | 1.48  (0.79 to 2.77) |
|  | Yes | 144 | 29 |  |  |  |  |  |  |  |  |  |  |  |  |  |  |
| Any siblings | No | 206 | 45 | 0.76 | 1.08  (0.66 to 1.76) | 96  76 | 24  17 | 0.69 | 0.86  (0.42 to 1.76) | 206  164 | 75  51 | 0.28 | 0.79  (0.51 to 1.22) | 96  76 | 34  28 | 0.85 | 1.06  (0.57 to 1.99) |
|  | Yes | 164 | 38 |  |  |  |  |  |  |  |  |  |  |  |  |  |  |
| Mother smoked | No | 311 | 66 | 0.20 | 1.50  (0.80 to 2.81) | 152  20 | 33  8 | 0.08 | 2.40  (0.91 to 6.37) | 311  59 | 107  19 | 0.74 | 0.91  (0.50 to 1.64) | 152  20 | 53  9 | 0.38 | 1.53  (0.60 to 3.92) |
|  | Yes | 59 | 17 |  |  |  |  |  |  |  |  |  |  |  |  |  |  |
| **Household smoked** | No | **217** | **38** | **0.01** | **1.96**  **(1.20 to 3.21)** | **103**  **69** | **16**  **25** | **<0.01** | **3.09**  **(1.50 to 6.38)** | 217  153 | 71  55 | 0.52 | 1.15  (0.75 to 1.78) | 103  69 | 34  28 | 0.31 | 1.39  (0.74 to 2.61) |
|  | Yes | **153** | **45** |  |  |  |  |  |  |  |  |  |  |  |  |  |  |
| Any pets | No | 183 | 42 | 0.81 | 0.94  (0.58 to 1.54) | 83  89 | 20  21 | 0.94 | 0.97  (0.48 to 1.96) | 183  187 | 57  69 | 0.24 | 1.29  (0.84 to 1.99) | 83  89 | 25  37 | 0.12 | 1.65  (0.88 to 3.10) |
|  | Yes | 187 | 41 |  |  |  |  |  |  |  |  |  |  |  |  |  |  |
| Cats | No | 283 | 64 | 0.88 | 0.96  (0.54 to 1.71) | 136  36 | 31  10 | 0.53 | 1.30  (0.57 to 2.99) | 283  87 | 97  29 | 0.87 | 0.96  (0.58 to 1.59) | 136  36 | 45  17 | 0.12 | 1.81  (0.86 to 3.81) |
|  | Yes | 87 | 19 |  |  |  |  |  |  |  |  |  |  |  |  |  |  |
| Dogs | No | 268 | 60 | 0.97 | 1.01  (0.58 to 1.74) | 122  50 | 30  11 | 0.72 | 0.86  (0.39 to 1.90) | 268  102 | 91  35 | 0.95 | 1.02  (0.63 to 1.64) | 122  50 | 43  19 | 0.73 | 1.13  (0.57 to 2.23) |
|  | Yes | 102 | 23 |  |  |  |  |  |  |  |  |  |  |  |  |  |  |
| Rabbits | No | 355 | 79-82 | 0.40 | ~0.5  (~0.1 to ~2) | 162  10 | 37-40  <5 | 0.31 | ~0.3  (~0.1 to ~3) | 355  15 | 119-122  <5 | 0.54 | ~0.7  (~0.2 to ~2) | 162  10 | 58-61  <5 | 0.68 | ~0.8  (~0.2 to ~3) |
|  | Yes | 15 | <5 |  |  |  |  |  |  |  |  |  |  |  |  |  |  |
| **Rodents** | No | 349 | 77 | 0.49 | 1.41  (0.53 to 3.76) | 162  10 | 37-40  <5 | 0.77 | ~0.8  (~0.2 to ~4) | **349**  **21** | **114**  **12** | **0.03** | **2.75**  **(1.13 to 6.71)** | 162  10 | 56  6 | 0.12 | 2.84  (0.77 to 10.48) |
|  | Yes | 21 | 6 |  |  |  |  |  |  |  |  |  |  |  |  |  |  |
| **Birds** | No | 360 | 79-82 | 0.85 | ~0.9  (~0.2 to ~4) | 166  6 | 37-40  <5 | 0.58 | ~1.6  (~0.3 to ~10) | 360  10 | 120  6 | 0.09 | 3.00  (0.83 to 10.83) | **166**  **6** | **57**  **5** | **0.04** | **9.56**  **(1.09 to 83.81)** |
|  | Yes | 10 | <5 |  |  |  |  |  |  |  |  |  |  |  |  |  |  |
| In least deprived fifth | No | 265 | 66 | 0.07 | 0.58  (0.32 to 1.05) | 118  54 | 30  11 | 0.47 | 0.75  (0.34 to 1.64) | 265  105 | 96  30 | 0.16 | 0.70  (0.43 to 1.15) | 118  54 | 47  15 | 0.13 | 0.58  (0.29 to 1.17) |
|  | Yes | 105 | 17 |  |  |  |  |  |  |  |  |  |  |  |  |  |  |
| **In most deprived fifth** | No | **265** | **49** | **<0.01** | **2.11**  **(1.26 to 3.53)** | 130  42 | 29  12 | 0.41 | 1.39  (0.63 to 3.06) | 265  105 | 94  32 | 0.36 | 0.80  (0.49 to 1.30) | 130  42 | 49  13 | 0.43 | 0.74  (0.35 to 1.56) |
|  | Yes | **105** | **34** |  |  |  |  |  |  |  |  |  |  |  |  |  |  |
| Household damp | No | 273 | 60 | 0.62 | 1.15  (0.66 to 1.99) | 126  45 | 30  11 | 0.93 | 1.04  (0.47 to 2.29) | 273  94 | 96  29 | 0.45 | 0.82  (0.50 to 1.36) | 126  45 | 48  14 | 0.40 | 0.73  (0.35 to 1.52) |
|  | Yes | 94 | 23 |  |  |  |  |  |  |  |  |  |  |  |  |  |  |
| Household mould | No | 311 | 70 | 0.91 | 1.04  (0.53 to 2.04) | 140  32 | 33  8 | 0.86 | 1.08  (0.44 to 2.63) | 311  56 | 107  18 | 0.74 | 0.90  (0.49 to 1.66) | 140  32 | 50  12 | 0.85 | 1.08  (0.49 to 2.39) |
|  | Yes | 56 | 13 |  |  |  |  |  |  |  |  |  |  |  |  |  |  |
| High risk doctor diagnosed | No | 52 | 9 | 0.34 | 1.45  (0.67 to 3.11) | 22  150 | <5  37-40 | 0.24 | ~2  (~0.6 to ~8) | 52  318 | 15  111 | 0.39 | 1.32  (0.70 to 2.52) | 22  150 | 7  55 | 0.66 | 1.24  (0.48 to 3.23) |
|  | Yes | 318 | 74 |  |  |  |  |  |  |  |  |  |  |  |  |  |  |
| High risk self diagnosed | No | 33 | <5 | 0.15 | ~2  (~0.8 to ~7) | 15  157 | <5  37-40 | 0.14 | ~5  (~0.6 to ~40) | 33  337 | 6  120 | 0.0502 | 2.49  (0.999 to 6.20) | 15  157 | 5  57 | 0.82 | 1.14  (0.37 to 3.50) |
|  | Yes | 337 | 79-82 |  |  |  |  |  |  |  |  |  |  |  |  |  |  |
| Mother allergy self reported | No | 101 | 23 | 0.92 | 0.97  (0.56 to 1.68) | 42  130 | 8  33 | 0.40 | 1.45  (0.61 to 3.44) | 101  269 | 33  93 | 0.73 | 1.09  (0.67 to 1.77) | 42  130 | 14  48 | 0.67 | 1.17  (0.56 to 2.44) |
|  | Yes | 269 | 60 |  |  |  |  |  |  |  |  |  |  |  |  |  |  |
| Father allergy self reported | No | 36 | 5 | 0.20 | 1.90  (0.71 to 5.06) | 21  147 | <5  36-39 | 0.59 | ~1.4  (~0.4 to ~4) | 36  328 | 12  111 | 0.95 | 1.02  (0.49 to 2.12) | 21  147 | 6  55 | 0.43 | 1.49  (0.55 to 4.08) |
|  | Yes | 328 | 77 |  |  |  |  |  |  |  |  |  |  |  |  |  |  |
| Mother allergy doctor diagnosed | No | 147 | 34 | 0.79 | 0.94  (0.57 to 1.54) | 61  111 | 13  28 | 0.56 | 1.25  (0.59 to 2.63) | 147  223 | 48  78 | 0.64 | 1.11  (0.71 to 1.72) | 61  111 | 21  41 | 0.74 | 1.12  (0.58 to 2.15) |
|  | Yes | 223 | 49 |  |  |  |  |  |  |  |  |  |  |  |  |  |  |
| **Father allergy doctor diagnosed** | No | 190 | 36 | 0.09 | 1.54  (0.94 to 2.52) | **88**  **80** | **15**  **25** | **0.03** | **2.21**  **(1.07 to 4.59)** | **190**  **174** | **54**  **69** | **0.02** | **1.66**  **(1.07 to 2.56)** | 88  80 | 29  32 | 0.34 | 1.36  (0.72 to 2.55) |
|  | Yes | 174 | 46 |  |  |  |  |  |  |  |  |  |  |  |  |  |  |
| *Note: Small numbers have been masked to maintain anonymity. Independent variables showing significance to the 5% level are highlighted in bold typeface with grey background.* | | | | | | | | | | | | | | | | | |

**Table S8. Multivariate logistic regression analysis of factors affecting atopic outcomes**

| **Atopic Outcome** | **Endpoint Time** | **Type of analysis** | **Variables significant in univariate logistic regression analysis** | **Retained in final model** | **p-value** | **OR**  **(95CI lower, Upper)** | **Numbers**  **in analysis** | **-2 log likelihood** | **NR^2^** | **HL**  **(*x*^2^,df,p)** | **Predictions**  **(% correct)** |
| --- | --- | --- | --- | --- | --- | --- | --- | --- | --- | --- | --- |
|  | | | | | | | | | | | |
| Asthma stage 1 | 2 years | Intention to treat | **Breastfeeding** | **Yes** | **<0.01** | **0.38 (0.20 to 0.72)** | 306 | 253.29 | 0.05 | 0,0,- | 84.64 |
|  |  |  | Least deprived fifth | No |  |  |  |  |  |  |  |
|  |  |  | Most deprived fifth | No |  |  |  |  |  |  |  |
|  |  | | | | | | | | | | |
|  | 5 years | Intention to treat | **Father allergy DR diagnosed** | **Yes** | **0.03** | **1.86(1.08 to 3.21)** | 272 | 322.63 | 0.10 | 6.20,7,0.52 | 69.85 |
|  |  |  | Most deprived fifth | No |  |  |  |  |  |  |  |
|  |  |  | High risk self diagnosed | Yes | 0.08 | 3.84(0.83 to 17.65) |  |  |  |  |  |
|  |  |  | High risk Dr diagnosed | No |  |  |  |  |  |  |  |
|  |  |  | Breastfeeding | Yes | 0.09 | 0.62(0.36 to 1.07) |  |  |  |  |  |
|  |  |  | Smoking in household | Yes | 0.10 | 1.58(0.92 to 2.71) |  |  |  |  |  |
|  |  |  | Father allergy self reported | No |  |  |  |  |  |  |  |
|  |  |  | | | | | | | | | |
|  |  | *Per protocol* | **Father allergy DR diagnosed** | **Yes** | **0.01** | **2.48(1.22-5.02)** | 168 | 197.40 | 0.15 | 1.58,3,0.66 | 70.24 |
|  |  |  | **Smoking in household** | **Yes** | **0.03** | **2.16(1.09-4.23)** |  |  |  |  |  |
|  |  |  | High risk Dr diagnosed | No |  |  |  |  |  |  |  |
|  |  |  | Smoking by mother | No |  |  |  |  |  |  |  |
|  |  |  | High risk self diagnosed | No |  |  |  |  |  |  |  |
|  | | | | | | | | | | | |
| Asthma stage 2 | 2 years | Intention to treat | **Breastfeeding** | **Yes** | **<0.01** | **0.27(0.11 to 0.66)** | 306 | 159.80 | 0.10 | 0.29,2,0.87 | 91.83 |
|  |  |  | Most deprived fifth | No |  |  |  |  |  |  |  |
|  |  |  | Least deprived fifth | Yes | 0.11 | 0.41(0.13 to 1.23) |  |  |  |  |  |
|  |  | | | | | | | | | | |
|  | 5 years | Intention to treat | Most deprived fifth | No |  |  | 276 | 282.29 | 0.05 | 1.44,2,0.49 | 77.90 |
|  |  |  | **Smoking in household** | **Yes** | **0.04** | **1.83(1.02 to 3.29)** |  |  |  |  |  |
|  |  |  | Breastfeeding | Yes | 0.06 | 0.57(0.32 to 1.02) |  |  |  |  |  |
|  |  |  | | | | | | | | | |
|  |  | *Per protocol* | **Smoking in household** | **Yes** | **<0.01** | **3.77(1.70-8.33)** | 168 | 162.40 | 0.18 | 1.85,6,0.93 | 76.79 |
|  |  |  | **Father allergy DR diagnosed** | **Yes** | **0.04** | **2.24(1.04-4.85)** |  |  |  |  |  |
|  |  |  | **Treatment group** | **Yes** | **<0.01** | **3.33(1.47 - 7.14)** |  |  |  |  |  |
|  | | | | | | | | | | | |
| Asthma stage 3 | 5 years | Intention to treat | **Father allergy DR diagnosed** | **Yes** | **0.01** | **2.62(1.33 to 5.16)** | 364 | 252.33 | 0.08 | 2.20,6,0.90 | 87.91 |
|  |  |  | Smoking in household | Yes | 0.07 | 1.83(0.95 to 3.54) |  |  |  |  |  |
|  |  |  | Least deprived fifth | Yes | 0.12 | 0.50(0.21 to 1.19) |  |  |  |  |  |
|  | | | | | | | | | | | |
| Eczema | 2 years | Intention to treat | **Father allergy DR diagnosed** | **Yes** | **0.01** | **1.90(1.18 to 3.06)** | 402 | 422.63 | 0.06 | 0.36,2,0.84 | 76.62 |
|  |  |  | **Daycare attendance** | **Yes** | **0.02** | **1.76(1.10 to 2.82)** |  |  |  |  |  |
|  |  |  | Birds | Yes | 0.06 | 3.07(0.95 to 9.98) |  |  |  |  |  |
|  |  |  | | | | | | | | | |
|  |  | *Per protocol* | **Father allergy DR diagnosed** | **Yes** | **0.01** | **2.36(1.19-4.67)** | 191 | 204.58 | 0.10 | 0,0,- | 75.92 |
|  |  |  | **Birds** | **Yes** | **0.02** | **7.96(1.44-43.92)** |  |  |  |  |  |
|  |  | | | | | | | | | | |
|  | 5 years | Intention to treat | **Father allergy DR diagnosed** | **Yes** | **0.05** | **1.56(1.00 to 2.43)** | 364 | 456.68 | 0.03 | 0.02,1,0.90 | 66.76 |
|  |  |  | Rodents | Yes | 0.05 | 2.46(1.00 to 6.09) |  |  |  |  |  |
|  | | | | | | | | | | | |
| *Explanatory notes: Variables significant at the 5% level from logistic regression univariate analysis were entered into a multivariate model in order of ascending univariate p-value using backward LR method, where there were more than one significant variables for the outcome. Variables retaining significance in the final model are highlighted in* ***bold*** *typeface. NR^2^=Nagelkerke R Square. HL = Hosmer and Lemeshow test* | | | | | | | | | | | |

**Table S9. Comparison of electronic data to fieldwork data (2 yr outcomes)**

| **Atopic outcome** | **Participant compliance** | **Cross tabulation of numbers in analysis** | | | | | **Agreement measure – Cohen’s Kappa**  (Asymp. Std. Error^a^ ,  Approx. T^b^,  Approx. Sig.) |
| --- | --- | --- | --- | --- | --- | --- | --- |
|  | | | | Fieldwork data | |  | |
| **Asthma** | Any |  |  | No | Yes | Total | **0.53**  (0.09, 9.48, <0.01) |
|  |  | Electronic Follow-up | No | **285** | **10** | 295 |  |
|  |  |  | Yes | **10** | **13** | 23 |  |
|  |  | (2yr) | Total | 295 | 23 | 318 |  |
|  |  | | | | | | |
|  | Good |  |  | No | Yes | Total | **0.54**  (0.13, 7.00, <0.01) |
|  |  | Electronic Follow-up | No | **141** | **<5** | 142-146 |  |
|  |  |  | Yes | **7** | **6** | 13 |  |
|  |  | (2yr) | Total | 148 | 8 | 156 |  |
|  | | | | | | | |
| **Eczema** | Any |  |  | No | Yes | Total | **0.47**  (0.06, 8.48, <0.01) |
|  |  | Electronic Follow-up | No | **201** | **40** | 241 |  |
|  |  |  | Yes | **27** | **53** | 80 |  |
|  |  | (2yr) | Total | 228 | 93 | 321 |  |
|  |  | | | | | | |
|  | Good |  |  | No | Yes | Total | **0.49**  (0.08, 6.15, <0.01) |
|  |  | Electronic Follow-up | No | **97** | **20** | 117 |  |
|  |  |  | Yes | **12** | **27** | 39 |  |
|  |  | (2yr) | Total | 109 | 47 | 156 |  |
|  | | | | | | | |
| **Antibacterials** | Any |  |  | No | Yes | Total | **0.26**  (0.04, 5.65, <0.01) |
|  |  | Electronic Follow-up | No | **154** | **116** | 270 |  |
|  |  |  | Yes | **38** | **100** | 138 |  |
|  |  | (2yr) | Total | 192 | 216 | 408 |  |
|  |  | | | | | | |
|  | Good |  |  | No | Yes | Total | **0.27**  (0.07, 4.03, <0.01) |
|  |  | Electronic Follow-up | No | **68** | **52** | 120 |  |
|  |  |  | Yes | **20** | **54** | 74 |  |
|  |  | (2yr) | Total | 88 | 106 | 194 |  |
|  | | | | | | | |
| *Explanatory notes: Cohen’s Kappa comparison for PROBAT electronic follow-up results versus fieldwork data. Agreement classifications: 0.00-0.20 slight, 0.21-0.40 fair, 0.41-0.60 moderate, 0.61–0.80 substantial, 0.81–0.99 almost perfect, 1.0 perfect (Landis and Koch, 1977). Asthma defined as >1 prescription of short acting beta 2 agonist (SBA) or = 1 prescription of SBA + GP asthma diagnosis.* | | | | | | | |

**Table S10. Sensitivity Analysis**

| **Atopic Outcome** | **E-Follow up time** | **Type of analysis** | **Adjusted effect of probiotics, plus other significant variables** | **P value** | **Odds Ratio**  **(95% CI lower, Upper)** |
| --- | --- | --- | --- | --- | --- |
|  | | | | | |
| Asthma stage 1 | 2 years | Intention to treat | **Probiotics**  Breastfeeding | **0.83**  0.01 | **0.93** (0.47, 1.83)  0.39 (0.20, 0.77) |
|  |  | Per protocol | **Probiotics**  Breastfeeding | **0.21**  0.04 | **1.82** (0.72, 4.61)  0.37 (0.15, 0.93) |
|  |  | | | | |
|  | 5 years | Intention to treat | **Probiotics**  Father allergy | **0.45**  <0.01 | **0.84** (0.59, 1.32)  1.97 (1.25, 3.08) |
|  |  | Per protocol | **Probiotics**  Father allergy  Smoking in household | **0.18**  <0.01  0.02 | **1.60** (0.81, 3.18)  3.11 (1.58, 6.13)  2.28 (1.14, 4.54) |
|  | | | | | |
| Asthma stage 2  (‘Primary’ Definition of Asthma in the study) | 2 years | Intention to treat | **Probiotics**  Breastfeeding | **0.06**  0.01 | **2.56** (0.96, 6.84)  0.25 (0.09, 0.65) |
|  |  | Per protocol | **Probiotics** | **0.01** | **5** (1.5, 15) |
|  |  | | | | |
|  | 5 years | Intention to treat | **Probiotics**  Smoking in household | **0.53**  0.01 | **1.17** (0.72, 1.92)  1.98 (1.21, 3.25) |
|  |  | Per protocol | **Probiotics**  Father Allergy  Smoking in household | **<0.01**  0.04  <0.01 | **3.33** (1.47, 7.1)  2.24 (1.04, 4.85)  3.77 (1.70, 8.33) |
|  | | | | | |
| Asthma stage 3 | 2 years | Intention to treat | **Probiotics** | **0.10** | **0.41** (0.14, 1.2) |
|  |  | Per protocol | **Probiotics** | **0.69** | **0.70** (0.11, 4.26) |
|  |  | | | | |
|  | 5 years | Intention to treat | **Probiotics**  Father allergy | **0.99**  0.01 | **1.00** (0.53, 1.88)  2.62 (1.34, 5.13) |
|  |  | Per protocol | **Probiotics**  Smoking in house | **0.03**  <0.01 | **3.35** (1.12, 9.98)  5.94 (1.92, 18.4) |
|  | | | | | |
| Asthma stage 4 | 2 years | Intention to treat | **Probiotics** | **0.09** | *Not applicable due to zero count* |
|  |  | Per protocol | **Probiotics** | **0.33** | *Not applicable due to zero count* |
|  |  | | | | |
|  | 5 years | Intention to treat | **Probiotics** | **0.34** | **0.50** (0.12, 2.04) |
|  |  | Per protocol | **Probiotics** | **0.69** | **0.69** (0.11, 4.24) |
|  | | | | | |
| Asthma stage 5 | 2 years | Intention to treat | **Probiotics** | **0.69** | **1.37** (0.30, 6.18) |
|  |  | Per protocol | **Probiotics** | **0.54** | **2.13** (0.19, 23.9) |
|  |  | | | | |
|  | 5 years | Intention to treat | **Probiotics**  Smoking in house | **0.69**  0.02 | **1.19**( 0.51, 2.79)  2.87 (1.18, 6.95) |
|  |  | Per protocol | **Probiotics**  Smoking in house | **0.17**  0.01 | **2.62** (0.67,10.3)  18.71 (2.3,155) |
|  | | | | | |
| Eczema | 2 years | Intention to treat | **Probiotics**  Father allergy  Daycare attendance | **0.20**  0.01  0.01 | **0.72** (0.44, 1.19)  0.90 (1.14, 3.15)  2.00 (1.21, 3.30) |
|  |  | Per protocol | **Probiotics**  Father allergy  Keep birds in house | **0.48**  0.02  0.03 | **0.77** (0.37-1.60)  2.40 (1.14, 5.04)  7.23 (1.23, 42.6) |
|  |  | | | | |
|  | 5 years | Intention to treat | **Probiotics**  Father allergy DR diagnosed | **0.80**  0.02 | **0.94** (0.61, 1.46)  1.66 (1.07, 2.57) |
|  |  | Per protocol | **Probiotics**  Keep birds in house | **0.67**  0.04 | **0.87**(0.46,1.64)  9.60(1.09,84.22) |
|  | | | | | |
| *Explanatory notes: The effect of treatment estimated in logistic regression model adjusting for any other background variables found significant at 5% level.* | | | | | |

**Further information is available from the authors.**
